# Effectiveness of gliclazide MR 60 mg in the management of type 2 diabetes: analyses from the EASYDia trial

**DOI:** 10.1186/s13098-018-0331-8

**Published:** 2018-04-10

**Authors:** Lawrence A. Leiter, Marina V. Shestakova, Ilhan Satman

**Affiliations:** 1 Division of Endocrinology & Metabolism, Li Ka Shing Knowledge Institute of St. Michael’s Hospital, University of Toronto, 61 Queen Street East, #6121Q, Toronto, ON M5C 2T2 Canada; 2 Institute of Diabetes Mellitus, Endocrinology Research Centre and M.I. Sechenov First Moscow State Medical University, Moscow, Russian Federation; 3 Division of Endocrinology and Metabolism, Department of Internal Medicine, Istanbul Faculty of Medicine, Istanbul University, Istanbul, Turkey

**Keywords:** Type 2 diabetes, Gliclazide, Glycemic control, Tolerability, Real-world

## Abstract

**Background:**

Although the number of antihyperglycemic agents has expanded significantly, sulfonylureas (in particular gliclazide) remain an important option because of a variety of patient and health system factors. The large, real world, observational, and international EASYDia trial evaluated the effectiveness of gliclazide modified release (MR) 60 mg in individuals with type 2 diabetes with a broad range of diabetes history, body mass index (BMI) and background antihyperglycemic treatment.

**Methods:**

A total of 7170 participants from eight countries, age ≥ 35 years with HbA1c ≥ 7.5% and not treated with insulin, were prescribed 30–120 mg of gliclazide MR 60 mg once daily. HbA1c goals were individualized and dosing uptitrated, as required, over the 6-month long study. In this post hoc subanalysis, efficacy endpoints were analyzed according to stratified baseline HbA1c levels, weight and glucose-lowering regimens. Episodes of hypoglycemia requiring assistance were documented.

**Results:**

At baseline, mean age was 58.9 years, HbA1c 8.8%, BMI 30.1 kg/m^2^, and diabetes duration 5.1 years. At study end, clinically significant HbA1c improvements (mean change − 1.78%) were noted across all baseline HbA1c strata (> 7.0 to ≤ 8.0%, > 8.0 to ≤ 9.0%, > 9.0 to ≤ 10.0%, and > 10.0%), BMI classifications (18.5 to < 25.0, 25.0 to < 30.0, and ≥ 30.0 kg/m^2^), and regardless of the original diabetes treatment regimen (*P *< 0.001 in all cases). In contrast to the subgroups with BMI 25.0–30.0 and ≥ 30.0 kg/m^2^ that registered weight losses of 0.9 and 2.2 kg, respectively (*P *< 0.001 vs. baseline weight); the BMI 18.5–24.9 kg/m^2^ subgroup gained a mean 0.5 kg (*P *< 0.02 vs. baseline weight). Severe hypoglycemic events were rare (0.06%).

**Conclusions:**

Progressive gliclazide MR 60 mg uptitration was well tolerated and lowered HbA1c across a broad range of HbA1c, BMI and background glucose-lowering therapy. Weight loss was noted when BMI was ≥ 25.0 kg/m^2^. Individuals with the highest baseline HbA1c and BMI experienced the greatest HbA1c and weight improvements.

*Trial registration* ISRCTN Registry ISRCTN00943368 on 1st July 2011

## Background

The Action in Diabetes and Vascular Disease: Preterax and Diamicron Modified Release Controlled Evaluation (ADVANCE) study [1–3] demonstrated that progressive uptitration of a modified release (MR) formulation of gliclazide as part of an intensive treatment regimen provides consistent glycemic control associated with long-term benefits on the combined microvascular and macrovascular endpoint. Despite the availability of newer classes of antihyperglycemic agents, sulfonylureas (in particular gliclazide) still retain an important position in diabetes management because of considerations around familiarity, guideline recommendations, cost and coverage [4–12].

With the overarching goal of improving adherence, the once daily (QD) gliclazide MR 60 mg formulation is available as a scored tablet to allow for convenient titration. The effectiveness and tolerability of this gliclazide tablet were evaluated in the real-world ObsErvationAl Study to analYse titration of Diamicron MR 60 mg in daily clinical practice in a large population with uncontrolled type 2 diabetes (EASYDia) study. Following 6 months of progressive uptitration, individuals in the EASYDia cohort experienced significantly improved glycemic control, mild weight loss and rare events of hypoglycemia [13]. We describe herein the temporal and dose-associated efficacy of gliclazide MR 60 mg, how efficacy varied with the participants’ baseline features, and the potential clinical implications of these observations.

## Methods

### Study conduct and population

EASYDia was an international, open-label, nonrandomized, non-comparative observational study (ISRCTN00943368) that was conducted according to the standards and principles of the Declaration of Helsinki. Ethics approval was site specific and written informed consent obtained at or before the baseline visit.

Screening took place from July 2011 to February 2014 at 596 sites in eight countries (Armenia, Georgia, Lebanon, Malaysia, Russia, Slovenia, Switzerland and Turkey). Potential participants were required to fulfill all of the following criteria: ≥ 35 years old with type 2 diabetes, HbA1c ≥ 7.5%, and either treatment-naïve or using non-insulin antihyperglycemic therapies. Individuals who were pregnant or breast feeding, exhibited hypersensitive reactions to sulfonylureas, displayed severe hepatic or renal failure (creatinine clearance < 30 mL/min), were taking miconazole, demonstrated contraindication to gliclazide, presented with uncontrolled and clinically significant disease or known malignancy, had a high probability of non-adherence to the EASYDia study expectations were excluded.

Individuals entered into the study were prescribed 30–120 mg gliclazide MR 60 mg QD by their physicians as first line, add-on or a switch from a previous oral glucose-lowering regimen [13]. Dosing was capped at 120 mg QD and uptitration, initiated at the discretion of the investigators, was driven by fasting plasma glucose (FPG) levels measured at months 1, 2, and 3. There were provisions to introduce another oral antihyperglycemic agent should glycemic control remain sub-optimal with gliclazide 120 mg QD. HbA1c goals were personalized and the final visit occurred 6 months after study initiation.

### Endpoints

We report herein the efficacy and safety outcomes of gliclazide MR therapy according to the participants’ baseline features and background glucose-lowering regimens. The primary efficacy endpoint and secondary efficacy endpoints that included treatment doses (daily average and temporal changes) as well as HbA1c improvements and the percentage of participants achieving an HbA1c of ≤ 7.0 and ≤ 6.5% at study end have been reported [13].

### Data collection

In this post hoc subanalysis, data collected at baseline, month 3 and the end of the study were stratified according to initial HbA1c (> 7.0 to ≤ 8.0%, > 8.0 to ≤ 9.0%, > 9.0 to ≤ 10.0%, and > 10.0%), body mass index (BMI 18.5 to < 25.0, 25.0 to < 30.0 and ≥ 30.0 kg/m^2^) and prior glucose-lowering regimens. With regards to hypoglycemia, only severe episodes were collected on the case report forms. Severe hypoglycemic incidents were classified as those associated with transient central nervous system dysfunction without other apparent cause, in which the individual was unable to treat him/herself and required assistance from another party.

### Statistical analyses

Demographic data and other baseline characteristics are reported for the included set. FPG and HbA1c information were derived from the full analysis set [FAS; participants who had taken at least one dose of the study treatment that they had been prescribed and had at least one baseline value and one post-baseline value of FPG (or HbA1c) on file]. Weight results were calculated from the safety set. Unless otherwise stated, data are presented as mean (standard deviation, SD). Changes from the corresponding baseline values were assessed with a Wilcoxon signed rank test and two-sided 95% confidence interval. Statistical significance was set at an unadjusted *P* value of < 0.05. Statistical analyses were conducted with SAS version 9.1.

## Results

### Baseline characteristics of the EASYDia cohort

The baseline characteristics of the EASYDia cohort (N = 7170) have been reported [13]. Details specific to the baseline HbA1c- and BMI-stratified populations are shown in Table 1. In short, about two-fifths of the EASYDia participants were men; baseline age of the cohort was 58.9 (10.6) years, BMI 30.1 (5.0) kg/m^2^, FPG 10.2 (2.8) mmol/L, and HbA1c 8.8 (1.3)%. Over 44% of the EASYDia participants had a baseline BMI that was 30 kg/m^2^ or greater while 12 and 42% had documented BMI values of 18.5 to 24.9 and 25.0 to < 30 kg/m^2^, respectively at baseline. Approximately two-thirds of the participants had a baseline HbA1c level that was > 8.0%. About half of the cohort was newly diagnosed rendering the baseline diabetes duration at 5.1 (4.4) years. Metformin was the most commonly used oral antihyperglycemic agent at study entry. When considering pre-study antihyperglycemic therapy profiles, most of the participants were either drug naïve or treated with metformin monotherapy; very few were being managed with dual therapy that included either a sulfonylurea or a DPP-4i in combination with metformin.

**Table 1:** Demographics of the EASYDia cohort based on pre-specified baseline body mass index and HbA1c stratification

	EASYDia cohort (N = 7170)
Demography	
Men^a^	2949 (41.5)
Age (years)*	58.9 (10.6)
Baseline body mass index (kg/m^2^)^b^	
18.5 to < 25.0	838 (11.9)
25.0 to < 30.0	3029 (42.9)
≥ 30.0	3191 (45.2)
Baseline HbA1c (%)^c^	
> 7.0 to ≤ 8.0	1765 (17.4)
> 8.0 to ≤ 9.0	2074 (47.1)
> 9.0% to ≤ 10.0	809 (18.4)
> 10.0	758 (17.2)

Data are presented as n (%) or *mean (standard deviation)

^a^The gender of 63 (0.88%) participants were not reported; ^b^ Data collected from the safety set; ^c^ Data collected from the full analysis set

### Effectiveness of gliclazide MR

Incremental dosing of gliclazide MR, over a period of 6 months (actual mean treatment duration in the FAS was 5.6 ± 1.1 months) was previously reported to be associated with temporal improvements in glycemic control across the EASYDia cohort [13]. Indeed, these benefits were evident within 3 months of study initiation. At months 3 and 6, the FPG levels for the entire cohort averaged 7.1 (1.7) and 6.8 (1.7) mmol/L, respectively; this represented an improvement of 3.4 (2.8) mmol/L (*P *< 0.001) over the 6-month study window. HbA1c at month 3 and study end were 7.3 (2.5)% and 6.9 (0.8)%, respectively which translated to a 1.82 (1.25) % absolute reduction from baseline (*P *< 0.001) at study end.

**Fig. 1:**
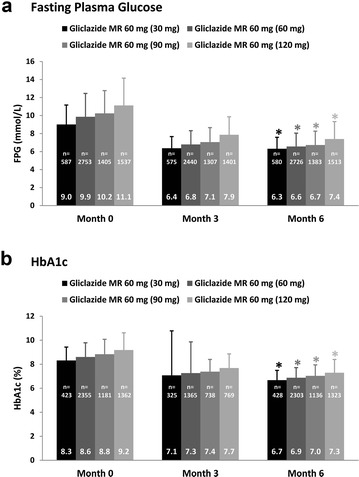
Temporal and dose-associated improvements in (**a**) FPG and (**b**) HbA1c stratified according to the month 6 gliclazide MR dose. *P < 0.001 for the difference between corresponding baseline and month 6 FPG and HbA1c values. FPG and HbA1c values are presented as mean (SD)

Figure 1 shows the temporal changes in FPG and HbA1c when the EASYDia participants were stratified according to the dose of gliclazide MR prescribed at month 6. Notably, the sub-cohort assigned the highest dose of gliclazide (120 mg QD) at month 6 had, at the beginning of the study, the highest mean FPG and HbA1c of 11.1 mol/L and 9.2%, respectively. The mean differences in baseline FPG and HbA1c values between those taking gliclazide MR 30 mg and gliclazide MR 120 mg at month 6 were 2.1 mmol/L and 0.9%, respectively. At month 3, comparisons between the same groups yielded smaller differences of 1.5 mmol/L for FPG and 0.6% for HbA1c. Although the gap between the two groups, at month 6, for FPG narrowed further to 1.1 mmol/L, that for HbA1c did not show further improvement. The same declining trends were observed when mean FPG and HbA1c values were compared between the month 6 gliclazide MR 60 mg and gliclazide MR 120 mg groups as well as the month 6 gliclazide MR 90 mg and gliclazide MR 120 mg arms (Fig. 1). These findings would suggest that a strategy of progressive uptitration of the gliclazide MR formulation is associated with improvements in glycemic control.

**Fig. 2:**
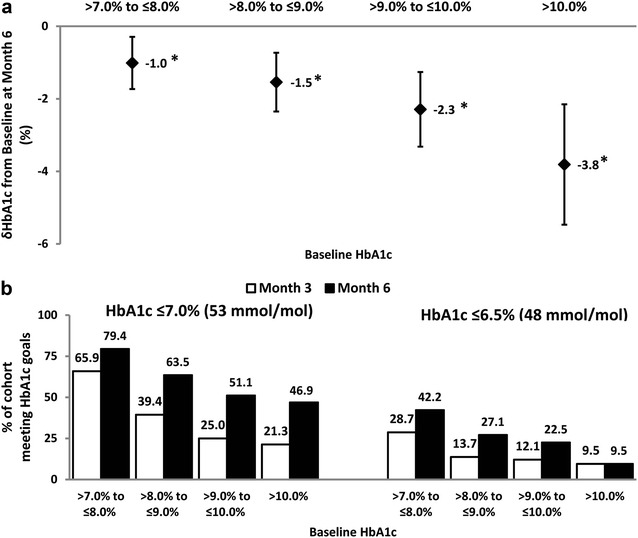
HbA1c lowering efficacy of gliclazide MR (**a**) at month 6 as stratified by baseline HbA1c and (**b**) as defined by the achievement of HbA1c ≤ 7.0 and ≤ 6.5% at months 3 and 6. n = 1765 for the “ > 7.0 to ≤ 8.0%” group; n = 2074 for the “ > 8.0 to ≤ 9.0%” group; n = 809 for the “ > 9.0 to  ≤ 10.0%” group and n = 758 for the “ > 10.0%” group. *P < 0.001 for the difference between corresponding baseline and month 6 HbA1c values. HbA1c data are presented as mean (SD)

Analysis of the data according to baseline HbA1c revealed significant HbA1c reductions across the board (*P* < 0.001). Notably, the most impactful HbA1c lowering was observed in the group with the highest baseline HbA1c levels (> 10.0%) and the least in the stratum with the lowest baseline HbA1c values (> 7.0– ≤ 8.0%) (Fig. 2a). Improvements in glycemic control were evident as early as month 3 in all four baseline HbA1c strata (Fig. 2b). Of note, at month 6, almost half of those in the baseline HbA1c > 10.0% group had achieved an HbA1c of ≤ 7.0%. Of further interest is the observation that at month 3, all four baseline HbA1c sub-groups included individuals whose HbA1c had declined to below 6.5% (Fig. 2b).

To determine if the glycemic improvements observed with gliclazide MR therapy varied across BMI status, HbA1c improvements were analyzed according to the pre-specified baseline BMI categories. Notably, a consistent and significant HbA1c improvement of 1.80% (*P *< 0.001) was noted for each of the BMI subgroups (18.5 to < 25.0, 25.0 to < 30.0 and ≥ 30.0 kg/m^2^) at month 6.

**Fig. 3:**
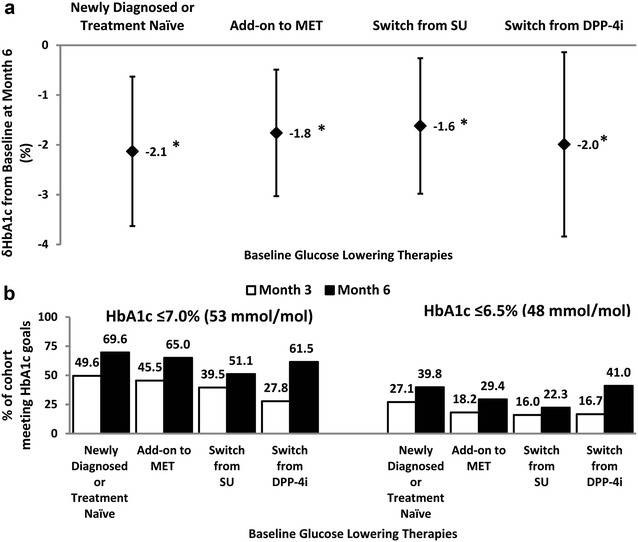
HbA1c lowering efficacy of gliclazide MR (**a**) at month 6 as stratified by baseline glucose lowering strategies and (**b**) as defined by the achievement of HbA1c ≤ 7.0 and ≤ 6.5% at months 3 and 6. n = 1142 for the “Newly Diagnosed or Treatment Naïve” group; n = 2550 for the “Add-on to MET” group; n = 819 for the “Switch from SU” group and n = 249 for the “Switch from DPP-4i” group. *P < 0.001 for the difference between corresponding baseline and month 6 HbA1c values. HbA1c data are presented as mean (SD). DPP-4i, dipeptidyl peptidase-4 inhibitor; *MET* metformin; *SU* sulfonylurea

Stratification according to pre-study diabetes treatment regimens, amongst which were switches from another sulfonylurea or a dipeptidyl peptidase-4 inhibitor (DPP-4i), revealed significant HbA1c improvements with gliclazide MR in all four strata examined (Fig. 3a; *P *< 0.001). Interestingly, the magnitude of the HbA1c improvement was very similar across the groups. It is worth noting that at month 3, nearly half of those who were newly diagnosed, treatment-naive or had been on metformin monotherapy at baseline had successfully achieved an HbA1c ≤ 7.0% with gliclazide MR add-on therapy (Fig. 3b). Additionally, at month 6, over half of those who had been switched from either another sulfonylurea or a DPP-4i met the HbA1c ≤ 7.0% goal; the more stringent HbA1c ≤ 6.5% target was attained at month 6 by more than two-fifths of those who were originally on a DPP-4i (Fig. 3b).

**Fig. 4:**
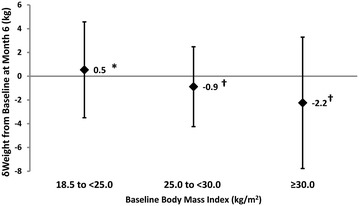
Weight lowering efficacy of gliclazide MR stratified according to calculated baseline body mass index (BMI). n = 838 for the “18.5–24.9 kg/m^2^” group; n = 3029 for the “25.0 to < 30 kg/m^2^” group; and n = 3191 for the “ ≥ 30 kg/m^2^” group. *P = 0.02 and ^†^*P *< 0.001 for the difference between corresponding baseline and month 6 weights. BMI data are presented as mean (SD)

The study closure weight of 81.7 (13.8) kg for the entire cohort represented a weight difference from baseline of − 1.3 (4.6) kg over 6 months. Of note, while the two subgroups with the higher BMI ranges (25.0 to < 30.0 and ≥ 30.0 kg/m^2^) registered statistically significant weight losses at month 6 (both *P *< 0.001 vs baseline weight), that with BMI 18.5–24.9 kg/m^2^ gained a mean of 0.54 (4.04) kg over the same time period (Fig. 4; *P* < 0.02 vs. baseline weight).

### Tolerability of gliclazide MR

Four of the EASYDia participants (0.06%) reported experiencing a total of five severe hypoglycemia episodes, 1 of which was determined to be unrelated to the study intervention. At the time that they experienced their hypoglycemic episodes, two of the three individuals whose hypoglycemic occurrences were suspected to be study drug-related were taking 90 mg gliclazide MR. All hypoglycemia events suspected to be related to the use and progressive uptitration of gliclazide MR were resolved promptly.

## Discussion

We report herein that incremental dosing of gliclazide MR 60 mg QD over 6 months was well tolerated and led to significant and clinically meaningful HbA1c reductions, as early as 3 months, in individuals with type 2 diabetes, not treated with insulin and with a broad range of baseline HbA1c. Additionally, HbA1c improvements of approximately 1.80% were documented in individuals regardless of whether they had a healthy BMI or if their BMI was 25.0 kg/m^2^ and above. Of note, baseline BMI values of 25.0 kg/m^2^ and higher were associated with significant weight losses. As seen with many antihyperglycemic agents, participants with the highest baseline HbA1c levels and BMI experienced the greatest HbA1c and weight improvements while the converse was true for those in the lowest baseline HbA1c and BMI strata.

The last 5 years have seen a steady stream of publications focused on the cardiovascular and renal safety as well as efficacy signals of novel diabetes medicines in large type 2 diabetes cohorts, the majority of whom had known cardiovascular disease [14–23]. While the results of these megatrials have generated much excitement albeit alongside some concerns about the newer classes of antihyperglycemic agents, sulfonylureas, in particular gliclazide, remain an important treatment option for many physicians and patients when glucose levels remain elevated despite the maximum tolerable dose of metformin [4–12].

Aside from metformin, the only other oral antihyperglycemic agent cited in the 2017 version of the World Health Organization Model List of Essential Medicines is gliclazide [9]. Along the same vein, several national guidelines [6, 10, 11] currently position gliclazide as a second-line choice, one recommends early initiation of gliclazide MR or glimepiride [12] while another [4] suggests adding a sulfonylurea, a DPP-4i, pioglitazone or a dual combination of the three when maximally tolerated metformin monotherapy does not achieve the desired glycemic outcome. Of note, gliclazide MR is recommended and widely used for the management of diabetes during Ramadan [24–26], a period during which prolonged fasting can render glycemic management challenging. The recommendations of these professional groups clearly support the continual role of gliclazide MR in contemporary diabetes management.

Inasmuch as progressive uptitration of gliclazide MR over 6 months produced clinically meaningful improvements in glycemic control regardless of baseline HbA1c suggests that individuals with suboptimally controlled type 2 diabetes along much of the diabetes continuum could benefit from the gliclazide MR regimen described in this report. Of further interest are the additional HbA1c reductions noted in EASYDia participants who had been switched from either another sulfonylurea or a DPP-4i to gliclazide MR. It is often believed that sulfonylureas as a class are typically associated with weight gain while DPP-4i are generally considered to be weight neutral. The unwanted weight effects of the former and the relatively more desirable weight profile of the latter frequently drive prescription decisions since weight gain is not only a common comorbidity and concern of individuals with type 2 diabetes but may also contribute to diminished adherence and hence propagate deranged glycemic control. The EASYDia subgroup with baseline BMI values of 25.0 kg/m^2^ to less than 30.0 kg/m^2^ lost a mean of 0.88 kg over 6 months while that with a BMI 30.0 kg/m^2^ and greater experienced a mean weight loss of 2.2 kg over the same time span. These observations might be considered unexpected since, as mentioned earlier, sulfonylureas have on average been linked with weight gain. That said, individuals who were newly diagnosed with type 2 diabetes made up almost one-fifth of the EASYDia cohort and given the early stages of their disease, these participants may have been more motivated to make lifestyle changes. Accordingly, we cannot discount the possibility that at least some of the weight benefits observed in the EASYDia program was in fact resultant of behavioural modifications. Regardless, the gliclazide MR regimen described herein may be a doubly favourable option for those with uncontrolled type 2 diabetes and a BMI that is above the healthy range. In support of this school of thought, the ADVANCE trial showed an overall no increase in weight over a median of 5 years in the gliclazide-based intensive therapy arm despite the improved glycemic control [1]. Additionally, the STudy Evaluating vildAgliptin compareD to gliclazide in patients with type 2 diabetes FASTing during Ramadan (STEADFAST) trial reported improvements in glycemic efficacy alongside small weight losses (mean − 1.1 ± 0.2 kg) with gliclazide therapy [27]. Accordingly, the current findings extend those of previous reports by demonstrating that progressive uptitration optimizes the impact gliclazide MR has on glycemic control with potential modest weight benefits in those with elevated BMI values.

Risk of hypoglycemia remains an important treatment consideration and is a major barrier for medication adherence, thereby hindering achievement of glycemic targets. Severe hypoglycemic episodes were uncommon, even amongst participants with only modest elevations in glycemia, with only five incidents reported by four EASYDia participants (0.06%). On further evaluation, however, it was noted that only three of these individuals experienced a total of four events that were suspected to be gliclazide-related (0.04%). The uncommon occurrences of severe hypoglycemia echo the low risk for hypoglycemia (relative to other sulfonylureas) reported with gliclazide MR in recent retrospective cohort studies (*P *< 0.001 vs. glyburide) [28] and an earlier five-country observational study that monitored gliclazide- and other sulfonylurea-treated individuals undergoing Ramadan fasting [26]. Although a small meta-analysis of three randomized trials calculated comparable occurrences of hypoglycemia in individuals using gliclazide and DPP-4i while fasting during Ramadan [29], small observational studies have documented greater, comparable or lower risk of hypoglycemia for gliclazide relative to sitagliptin and vildagliptin [27, 30, 31].

The EASYDia study has several strengths and limitations. First, the large multinational real-world dataset was obtained from clinically diverse individuals who were positioned along the entire diabetes continuum thus capturing a close representation of the global diabetes population. Second, the non-regimented design and setting allowed for a more realistic documentation of the benefits and harms associated with intensive gliclazide MR uptitration. At the same time, the non-randomized nature of this observational study, lack of formal comparator, and relatively short duration of follow-up, should be noted. Furthermore, given the fact that many of the EASYDia participants had newly diagnosed diabetes, it is possible that they may have been more motivated to initiate and sustain recommendations for lifestyle recommendations. Of specific consideration in this sub-analysis, there were imbalances in the sample sizes of the sub-cohorts examined and these likely contributed to the larger outcome variability observed in the smaller groups and tighter ranges measured in those with more participants.

In conclusion, the large, contemporary, real-world EASYDia study has shown that progressive uptitration of gliclazide MR over 6 months is well tolerated in individuals with type 2 diabetes not treated with insulin and is associated with clinically meaningful HbA1c reductions across a broad range of HbA1c and clinical scenarios. Individuals with BMI 25.0 kg/m^2^ and higher also experienced significant weight loss. Importantly, the rarity of severe hypoglycemia events observed with the gliclazide treatment, diminishes an important concern routinely surrounding the sulfonylurea class. Overall, these data highlight that the described gliclazide MR regimen is an effective option for a wide variety of non-insulin treated individuals with diabetes and inadequate glycemic control.

## Abbreviations

ADVANCE: Action in Diabetes and Vascular Disease: Preterax and Diamicron Modified Release Controlled Evaluation
BMI: body mass index
EASYDia: ObsErvationAl Study to analYse titration of Diamicron MR 60 mg in daily clinical practice in a large population with uncontrolled type 2 diabetes
DPP-4i: dipeptidyl peptidase-4 inhibitor
FAS: full analysis set
FPG: fasting plasma glucose
MET: metformin
MR: modified release
QD: once daily
SD: standard deviation
SU: sulfonylurea
